# Self-Control in Intertemporal Choice and Mediterranean Dietary Pattern

**DOI:** 10.3389/fpubh.2018.00176

**Published:** 2018-06-15

**Authors:** María J. Muñoz Torrecillas, Salvador Cruz Rambaud, Taiki Takahashi

**Affiliations:** ^1^Department of Economics and Business, Universidad de Almería, Almería, Spain; ^2^Department of Behavioral Science, Center for Experimental Research in Social Sciences, Hokkaido University, Sapporo, Japan

**Keywords:** mediterranean diet, intertemporal choice, time discounting, impulsivity, self-control

## Abstract

**Background:** The Mediterranean Diet (hereinafter MD) is considered a healthy dietary pattern. Adherence to this pattern can be assessed by means of the KIDMED test by which individuals are assigned an index and classified into three groups of adherence to MD: high, medium, and low. In addition, impulsivity or impatience in intertemporal choice has been defined as a strong preference for small immediate rewards over large delayed ones.

**Objective:** This study examines the relationship between dietary habits, specifically Mediterranean dietary pattern, measured by the KIDMED index, and the exhibited impatience in intertemporal choices, by means of the parameter *k* (discount rate of the hyperbolic discount function).

**Methods:** A sample of 207 university students answered a questionnaire based on two tests: the KIDMED test, to assess the degree of adherence to MD, and an intertemporal choice questionnaire, to assess impatience or impulsivity. Individuals were grouped depending on their KIDMED score and then the discount rate or impulsivity parameter was calculated for each group.

**Results:** Discount rates were inversely related to the degree of adherence to MD. The values of overall *k* were 1.53, 1.91, and 3.71% for the groups exhibiting high, medium and low adherence to MD, respectively. We also found higher *k*-values for larger rewards (magnitude effect) in the three groups.

**Conclusion:** High adherence to MD is related to less steep time discounting, which implies less impulsivity (more self-control) or lower discount rates. Conversely, low adherence to MD is related to steeper time discounting, which implies impulsivity or higher discount rates. These findings could be used to identify the target population where policy interventions are needed in order to promote healthier diet habits.

## Introduction

The traditional Mediterranean diet is characterized by a high consumption of plant foods (vegetables, fruits, legumes, and cereals), of olive oil as the principal source of mono-unsaturated fat (low intake of saturated fat), intake of fish, low-to-moderate intake of dairy products, low consumption of meat and poultry, and wine consumed in low-to-moderate amounts, normally with meals (1). This dietary pattern is considered a balanced and varied diet which provides most of the recommended macronutrients in their correct proportion (2).

Adherence to the Mediterranean dietary pattern has been associated with a longer life-span, reduced risk of cardiovascular and/or cancer mortality and of neurodegenerative disease (3, 4). It is therefore important, from the point of view of health economics, to examine the relationship between adherence to the Mediterranean diet, one of the most typical healthy eating habits, and economic decision-making over time. Specifically, the main objective of this paper is to examine the relationship between the Mediterranean dietary pattern and the exhibited impatience in intertemporal choices, by means of the parameter *k* of the hyperbolic discount function.

Adherence to MD in Spain, Greece, Cyprus, and Italy among children and adolescents was surveyed by Grosso and Galvano (5). A survey in Italy was carried out by Cavaliere et al. (6), where a significant part of the analyzed population followed dietary patterns alien to the MD pyramid guidelines. This may be due to the increasing cost of many key foods of the Mediterranean diet, which has led people to westernize their diet in favor of less expensive, often unhealthy products (7). A historical review of the adherence to MD worldwide can be found in da Silva et al. (8), and a similar review, in Spain, in Bach-Faig et al. (9).

There are several studies which show that unhealthy eating habits are related to impatience in intertemporal choice [for a systematic review, see (10)]. From this, we can deduce the opposite scenario. Since the Mediterranean diet is a model of healthy eating (2, 11–14), it can be predicted that the Mediterranean diet habit is related to self-control in intertemporal choice. This is the hypothesis we are going to test in the empirical study described in detail in section Materials and Methods. One of the advantages of the present study is that the data was collected in the Spanish regions close to the Mediterranean Sea. Contrarily, if this type of investigation were conducted in other areas such as North America, it would be virtually impossible to collect data on genuine Mediterranean dietary habits.

It is often suggested that the methodologies developed to measure the impatience in intertemporal choice could be applied to other domains such as health states or dieting (15). In effect, the existing literature on this topic has been focused on the use of discounting tools when analyzing the decisions about smoking, exercising, dieting, saving, etc. at different points in time [see (16–18)]. In this context, our paper aims to analyze the influence of the score obtained in the questionnaires administered to measure the aforementioned individual characteristics (more specifically, dieting) on the intertemporal choice of monetary rewards. Specifically, we take into account the impatience or impulsivity shown by individuals in their choices by means of the *k*-parameter (discount rate) of a discount function. The higher the value of *k*, the more impatient the individual is said to be. On the other hand, the opposite behavior to impulsivity is self-control.

Eating habits and dietary intake are a public health concern. In health economic studies, relationships between eating habits/disorders and self-control in intertemporal choices have been examined [see (19) for a review]. In Davis et al. (20) the roles of impulsivity in the Iowa gambling task and temporal discounting are reported. Among heavy drinkers, impulsivity in temporal discounting (i.e., preference for sooner smaller rewards over larger later ones) contributes to dysregulated eating (21). In both obese and non-obese women, impulsivity in intertemporal choices has been associated with problematic eating behavior (22, 23). Also in obese children, self-control in intertemporal choice predicted weight loss (24). In the context of behavioral interventions for problematic eating behavior, mindful eating training reduced preference for smaller and more immediate food rewards over larger later ones (25). Contrarily, among female students, self-control in intertemporal choice may be a risk factor for anorexia nervosa (26). In bulimia nervosa, in contrast, impulsivity in intertemporal choice was reportedly exacerbated (27). Drug misuse is also a public health issue. Thus, Mole et al. (28) reported that impulsivity in intertemporal choice is a common-core impairment across problematic eating and substance misuse. Among working adults, fast-food consumption (unhealthy eating habits) has been associated with greater temporal discounting (29). From certain perspectives in behavioral economics, it has been argued that higher future time preferences (i.e., self-control in intertemporal choice) are related to a lower frequency of fast-food consumption (30).

Nevertheless, to the extent of our knowledge, no previous paper has examined the relations between MD (a model of healthy dietary habits) and time discounting, and therefore our study is the first to examine this relationship.

## Materials and methods

To test our hypothesis, we have used a questionnaire based on two different tests. First, the KIDMED test (11) has been used to evaluate the adherence to the Mediterranean Diet (MD), and second the questionnaire devised by Kirby et al. (31) has been implemented to obtain appropriate information about intertemporal choice. We also requested some demographic data (such as sex, age, education, and employment status).

### KIDMED test and intertemporal choice questionnaire

In order to assess the degree of adherence to MD, we have used the KIDMED test developed by Serra-Majem et al. (11). This test is a 16-item questionnaire regarding food habits (see Appendix 1, Part 1) in which the answers can be either affirmative or negative. The answers with a positive connotation in relation to the principles of the MD are assigned a value of +1, and those with a negative connotation, a value of −1. We thereby obtain the KIDMED index with a value within the range 0 to 12. On this basis, each participant is classified in one of the following groups according to his/her score in the test (2):

1. KIDMED index ≥ 8 means high adherence to MD (optimal Mediterranean diet).
2. KIDMED index from 4 to 7 implies medium adherence to MD (improvement needed to adjust intake to Mediterranean patterns).
3. KIDMED index ≤ 3 means poor adherence to MD (very low diet quality).

To assess impulsivity, we have taken into account the level of impatience exhibited in intertemporal choices, by means of the *k*-parameter (discount rate) of a hyperbolic discount function (32):

(1) $$\begin{array}{r} \text{SIR}=\frac{\text{LDR}}{1+kd},\;k>0, \end{array}$$

where SIR is the smaller, immediate reward, LDR is the larger, delayed reward, and *d* is the delay associated with LDR.

For this purpose, we have used the 27-item monetary choice questionnaire developed by Kirby et al. (31) (see Appendix 1, Part 2 in the Supplementary Materials). This questionnaire contains a set of 27 specific choices between an SIR and a LDR. There are three levels of reward size: small (from $25 to $35), medium (from $50 to $60), and large (from $75 to $85). It is thereby possible to calculate for each participant a separate *k* value for small, medium, and large delayed rewards. Therefore, the magnitude effect, which implies higher discount rates for smaller than for larger amounts (33–35), can be assessed.

To derive the discount rates from the responses of the individual questionnaires, we have used the automated scores for an Excel spreadsheet as obtained from Kaplan et al. (36) which applies and facilitates the scoring procedure to estimate the discount rate, and which is fully described in Kirby et al. (31) and Kaplan et al. (37).

### Sample

A total of 207 students at the Business School of the University of Almería (Spain) participated by answering the questionnaires. The participation was completely voluntary. Eleven questionnaires were discarded: seven were incomplete and four were rejected for reasons of lack of consistency which will be explained in the following subsection. Our final sample therefore consisted of 196 subjects. Regarding the composition of our sample, 55% of the participants were men and 45% women, and the mean age was 22 years. Only 11% of the participants smoked, and only 15% combined their studies with paid employment.

### Procedure

We contacted the students at the Business School in their classes, using the last minutes of them, and prior agreement with their professors.

Before completing the questionnaire, students were informed that these would be completely anonymous and that we asked for their voluntary participation. Therefore, students who did not want to collaborate could leave the classroom or stay there and not to complete the questionnaires. Moreover, we asked them to respond with absolute sincerity and we gratefully acknowledged their participation in this research.

After collecting the data, we started by evaluating the KIDMED test and calculating the KIDMED index derived from the responses. According to their scores in the test, we classified the subjects in three groups: high, medium, and low adherence to MD, as explained in section KIDMED Test and Intertemporal Choice Questionnaire.

We subsequently introduced the intertemporal choices of each individual in the first group (high adherence to the MD) in the automated scores for an Excel spreadsheet using (36), obtaining the discount rates (*k*-values) for each individual and for the whole group. We proceeded in the same way with the other two groups (medium and low adherence to MD). This spreadsheet estimated for each individual the overall *k* value based on the answers to the 27 intertemporal choice questions. Moreover, according to the response patterns, a consistency score was determined for each participant. The recommendation of Kaplan et al. (36) is to examine more closely the individual-level patterns in cases where consistency scores are less than 75%, because it may indicate a lack of attention to the questionnaire. As we had four cases where individual consistency scores were less than 75%, we opted for their exclusion from the sample.

## Results

We had a final sample of 196 subjects divided into three groups depending on their adherence to MD: 29% of the subjects were included in the high-adherence group, 58% in the medium adherence group, and 13% in the low adherence group, according to their KIDMED index. These figures make sense if we take into account the place where the data were collected: Almería, a province in the south-east of Spain, on the Mediterranean coast. We have few people with low adherence to MD and a majority with medium and high adherence to MD. The composition of our sample, in terms of adherence to MD, is in line with other studies conducted among university students. Specifically Durà and Castroviejo (38) analyzed a sample of 570 university students with regard to the adherence to MD, using the KIDMED test. They had the following results: 28.4% of the students had high adherence to MD, 62.1% medium adherence to MD, and 9.5% low adherence to MD. As can be observed, the composition regarding adherence to MD in both samples is very similar.

Table 1 shows the mean, standard deviation (SD) and standard error of the mean (SEM) of the *k*-values for non-transformed, log-transformed and ln-transformed data corresponding to the three groups under study (high, medium, and low adherence to MD). These measures have been also discriminated by overall *k*-values, and *k*-values for the small, medium, and large magnitude sizes.

**Table 1:** Summary statistics for *k*-values in the three groups under study (high, medium and low adherence to MD).

	**High adherence**		**Medium adherence**		**Low adherence**	
	**Mean**	**SD**	**Mean**	**SD**	**Mean**	**SD**
Overall k	0.0153	0.0155	0.0191	0.0198	0.0371	0.0653
Small k	0.0264	0.0295	0.0356	0.0360	0.0481	0.0542
Medium k	0.0176	0.0193	0.0191	0.0194	0.0395	0.0556
Large k	0.0122	0.0234	0.0126	0.0172	0.0240	0.0562
Geomean k	0.0149	0.0143	0.0181	0.0184	0.0324	0.0524
log overall k	−2.1143	0.6229	−1.9606	0.5282	−1.7849	0.5352
log small k	−1.8775	0.6060	−1.7152	0.5888	−1.5413	0.4602
log medium k	−2.0763	0.6498	−1.9750	0.5491	−1.7220	0.5569
log large k	−2.3167	0.6431	−2.2218	0.5615	−2.1390	0.6086
log geomean k	−2.0902	0.5862	−1.9707	0.5103	−1.8007	0.4942
ln overall k	−4.8684	1.4342	−4.5145	1.2163	−4.1098	1.2323
ln small k	−4.3231	1.3954	−3.9494	1.3557	−3.5489	1.0597
ln Medium k	−4.7808	1.4963	−4.5475	1.2643	−3.9651	1.2824
ln large k	−5.3345	1.4807	−5.1159	1.2929	−4.9251	1.4014
ln geomean k	−4.8128	1.3497	−4.5376	1.1750	−4.1464	1.1380
Overall consistency	96.55%	3.98%	95.51%	4.52%	94.81%	4.90%
Consistency of small	98.66%	3.65%	98.53%	3.79%	97.78%	5.56%
Consistency of medium	98.66%	4.69%	97.74%	5.18%	97.33%	4.84%
Consistency of large	99.04%	4.31%	97.84%	5.11%	96.44%	6.19%
Composite consistency	98.79%	2.89%	98.03%	3.29%	97.19%	3.90%

*Geomean k is determined by taking the geometric mean of the resulting small, medium, and large k-values*.

**Figure 1:**
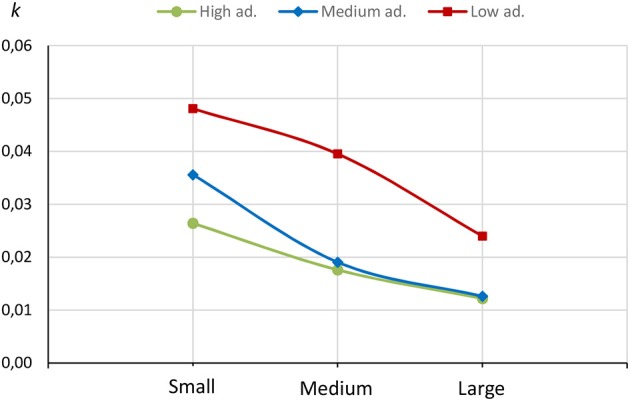
*k*-values for small, medium, and large delayed rewards in the three groups under study.

The obtained results reveal the existence of the magnitude effect in high, medium and low adherence to MD groups. As we can observe in Figure 1, higher discount rates (*k*) are applied to smaller delayed rewards, and vice versa (see Figure 1).

Table 1 also includes a measure of consistency which is a score which takes into account the instances of 0s (i.e., the choice of the SIR) prior to the given *k*-value, and instances of 1s (i.e., LDR) at and following the given *k*-value. This amount is then divided by 27 (in the case of the overall *k*) or 9 (in the case of a particular *k*-value).

Table 2 displays the Pearson correlations between the three possible pairs of transformed and non-transformed *k*-values. Likewise, it shows the intervals at a 95% confidence level.

**Table 2:** Pearson correlations for the three groups under study between small, medium and large *k*.

	**High adherence**			**Medium adherence**			**Low adherence**		
**Pairing**	**Correlation**	**Lower**	**Upper**	**Correlation**	**Lower**	**Upper**	**Correlation**	**Lower**	**Upper**
Small-Medium (*k*)	0.61	0.42	0.75	0.61	0.48	0.71	0.82	0.63	0.92
Small-Large (*k*)	0.16	−0.11	0.40	0.51	0.36	0.63	0.81	0.61	0.91
Medium-Large (*k*)	0.23	−0.03	0.46	0.64	0.51	0.73	0.95	0.90	0.98
Small-Medium (log *k*)	0.85	0.76	0.91	0.71	0.61	0.79	0.71	0.45	0.87
Small-Large (log *k*)	0.69	0.53	0.81	0.67	0.55	0.76	0.71	0.44	0.86
Medium-Large (log *k*)	0.82	0.71	0.89	0.78	0.69	0.84	0.80	0.58	0.91
Small-Medium (ln *k*)	0.85	0.76	0.91	0.71	0.61	0.79	0.71	0.45	0.87
Small-Large (ln *k*)	0.69	0.53	0.81	0.67	0.55	0.76	0.71	0.44	0.86
Medium-Large (ln *k*)	0.82	0.71	0.89	0.78	0.69	0.84	0.80	0.58	0.91

In Table 3, we have summarized the results for the three separate groups, taking into account only the overall *k*.

**Table 3:** Overall discount rates by groups and consistency of the results.

**Group**	**Overall *k* (%)**	**Overall consistency (%)**
High adherence to MD	1.53	96.55
Medium adherence to MD	1.91	95.51
Low adherence to MD	3.71	94.81

These results confirm our initial hypothesis: high adherence to MD is related to less steep time-discounting, or in other words, high adherence to MD implies lower discount rates, in this case 1.53% (the smallest discount rate of the three groups). Moreover, the group with low adherence to MD presents the highest discount rate: 3.71%.

As the rate of discount is commonly taken to indicate the level of impulsivity or impatience in intertemporal choices (39), we found that people with low adherence to MD exhibit more impatience than people with high adherence to MD. Moreover, a general deterioration of the MD among young people has been observed (5). In line with these observations, 58% of the subjects surveyed in our sample presented medium adherence to the MD, which means that efforts are needed to adjust their food consumption to Mediterranean patterns.

## Discussion

Our results are in line with those of other researchers [see (10)] who found higher discount rates in persons consuming unhealthy diets, and those who are overweight and obese.

Therefore, impatience could be an early indication of a problem of self-control which could result in overweigh, obesity or even other diseases (e.g., addiction), given that excessive discounting may be considered as a trans-disease process (40, 41). This finding could be used to identify the target population where policy interventions are needed in order to promote healthier diet habits.

Additionally, MD has been associated with a number of beneficial effects on cognitive functions. Among French people, Féart et al. (42) reported that MD has been associated with slower cognitive decline in elderly people (aged 65+). Recently, Luciano et al. (43) observed that MD adherence was associated with slower brain atrophy among a group of people in Scotland aged 73–76. This indicates that several components of MD may even protect neuronal structures in elderly people, which could help this sector of the population make better decisions. A recent meta-analysis study reported that MD adherence is related to several types of good memory and executive functions (44). For instance, episodic memory and global cognition were positively associated with MD adherence.

According to studies in cognitive psychology, self-control in time discounting is associated with working memory (45), attention (46), executive functions (47), episodic future thinking (48), and time perception (49). Future studies should examine which cognitive components are modulated by MD adherence.

As limitations of our study, we could include the size of the sample used to test our hypothesis or the fact that it includes only university students. Future research could replicate this study using a bigger and randomly selected sample. Nevertheless, our sample is in line with similar studies: in the 34 studies reviewed by McClelland et al. (19), the mean sample was 137, and only six papers out of 34 used a sample with more than 200 subjects.

Despite these possible limitations, the main strength of our study is the originality of the hypothesis submitted to test. To date, the relationship between healthy eating habits and intertemporal choice has not been extensively investigated, and specifically and to the extent of our knowledge, no previous paper has examined the relations between MD and time discounting.

More research is needed to support the relation between MD and time discounting. We aim to further investigate the relations between MD, BMI (body mass index) and time discounting.

## Ethics statement

Ethical review and approval was not required for this study in accordance with the national and institutional requirements. The University of Almería approved the collection of data among students who voluntarily agreed to answer the anonymous questionnaire.

## Author contributions

All authors listed have made a substantial, direct and intellectual contribution to the work, and approved it for publication.

### Conflict of interest statement

The authors declare that the research was conducted in the absence of any commercial or financial relationships that could be construed as a potential conflict of interest.
