# Supplementary material for: Self-Control in Intertemporal Choice and Mediterranean Dietary Pattern
Source: Front Public Health. 2018 Jun 15;6:176. doi: 10.3389/fpubh.2018.00176 (PMC6013565; doi:10.3389/fpubh.2018.00176)
Supplement: Supplementary file 1 [file Data_Sheet_1.pdf]

APPENDIX 1. QUESTIONNAIRE

These questionnaires will be **anonymous** and the data provided will not allow you to be identified. Please, respond to the questionnaire with complete sincerity.
We thank you in advance for your participation and the time you spend answering the questionnaire. **Thank you very much**.

GENERAL DATA

**1**	☐ Male<br>☐ Female	Date of birth:<br>______________	Place of birth (please write also the country if it is not Spain):<br>____________________
	In case you were not born in Spain, how long have you lived in our country?<br>_____ years		
	Height: _______ cm Weight: _______ kg		

**2**	Degree/Master in which you are enrolled:

**3**	¿Do you work?	☐ No, I only study<br>☐ Yes, Where?/Position:	________________________________

**4**	How many cigarettes do you usually smoke per day? _______<br>(In case you are non-smoker, please write “0”)

Part I

Please, think about what you usually do (most of the days) and answer yes or no.

	YES/NO
**1.** Do you take a fruit or fruit juice every day?	
**2.** Do you have a second fruit every day?	
**3.** Do you have fresh or cooked vegetables regularly once a day?	
**4.** Do you have fresh or cooked vegetables more than once a day?	
**5.** Do you consume fish regularly (at least 2 or 3 times per week)?	
**6.** Do you go more than once a week to a fast food restaurant (hamburger)?	
**7.** Do you like pulses and eat them more than once a week?	
**8.** Do you consume pasta or rice almost every day (5 or more per week)?	
**9.** Do you have cereals or grains (bread, etc.) for breakfast?	
**10.** Do you consume nuts regularly (at least 2 or 3 times per week)?	
**11.** Do you use olive oil at home?	
**12.** Do you skip breakfast?	
**13.** Do you have a dairy product for breakfast (yoghurt, milk, etc.)?	
**14.** Do you have commercially baked goods or pastries for breakfast?	
**15.** Do you take two yoghurts and/or some cheese (40 g) daily?	
**16.** Do you take sweets and candy several times every day?	

Part II

Instructions

In this part of the study, you will be asked **to make several decisions** about different amounts of money which you might gain at different points in time. Consider that the offer concerns real money. We are interested in which amount of money you would choose to receive if you were to be offered these choices **for real**.

There are no correct or incorrect choices. We are interested in which option you would really prefer. Decisions must be made based solely on your free will. Please, circle the option you would prefer for each of the following questions.

For each of the next 27 choices, please indicate which reward you would prefer: the smaller reward today or the larger reward in the specified number of days.

EXAMPLE:

0. Would you prefer:	$0 today,	or	$10 in 7 days?

To indicate your choice, please circle the option you would chose (as shown in the previous example) for each of the following questions.

1. Would you prefer:	$54 today,	or	$55 in 117 days?
2. Would you prefer:	$55 today,	or	$75 in 61 days?
3. Would you prefer:	$19 today,	or	$25 in 53 days?
4. Would you prefer:	$31 today,	or	$85 in 7 days?
5. Would you prefer:	$14 today,	or	$25 in 19 days?
6. Would you prefer:	$47 today,	or	$50 in 160 days?
7. Would you prefer:	$15 today,	or	$35 in 13 days?
8. Would you prefer:	$25 today,	or	$60 in 14 days?
9. Would you prefer:	$78 today,	or	$80 in 162 days?
10. Would you prefer:	$40 today,	or	$55 in 62 days?
11. Would you prefer:	$11 today,	or	$30 in 7 days?

12.Would you prefer:	$67 today,	or	$75 in 119 days?
13.Would you prefer:	$34 today,	or	$35 in 186 days?
14.Would you prefer:	$27 today,	or	$50 in 21 days?
15.Would you prefer:	$69 today,	or	$85 in 91 days?
16.Would you prefer:	$49 today,	or	$60 in 89 days?
17.Would you prefer:	$80 today,	or	$85 in 157 days?
18.Would you prefer:	$24 today,	or	$35 in 29 days?
19.Would you prefer:	$33 today,	or	$80 in 14 days?
20.Would you prefer:	$28 today,	or	$30 in 179 days?
21.Would you prefer:	$34 today,	or	$50 in 30 days?
22.Would you prefer:	$25 today,	or	$30 in 80 days?
23.Would you prefer:	$41 today,	or	$75 in 20 days?
24.Would you prefer:	$54 today,	or	$60 in 111 days?
25.Would you prefer:	$54 today,	or	$80 in 30 days?
26.Would you prefer:	$22 today,	or	$25 in 136 days?
27.Would you prefer:	$20 today,	or	$55 in 7 days?
